# Shining the light on clinical application of mesenchymal stem cell therapy in autoimmune diseases

**DOI:** 10.1186/s13287-022-02782-7

**Published:** 2022-03-07

**Authors:** Saade Abdalkareem Jasim, Alexei Valerievich Yumashev, Walid Kamal Abdelbasset, Ria Margiana, Alexander Markov, Wanich Suksatan, Benjamin Pineda, Lakshmi Thangavelu, Seyed Hossein Ahmadi

**Affiliations:** 1 Medical Laboratory Techniques Department, Al-Maarif University College, Al-Anbar-Ramadi, Iraq; 2 Department of Prosthetic Dentistry, Sechenov First Moscow State Medical University, Moscow, Russia; 3 Department of Health and Rehabilitation Sciences, College of Applied Medical Sciences, Prince Sattam Bin Abdulaziz University, Al Kharj, Saudi Arabia; 4 Department of Physical Therapy, Kasr Al-Aini Hospital, Cairo University, Giza, Egypt; 5 Department of Anatomy, Faculty of Medicine, Universitas Indonesia, Jakarta, Indonesia; 6 Master’s Programme Biomedical Sciences, Faculty of Medicine, Universitas Indonesia, Jakarta, Indonesia; 7 Dr. Soetomo General Academic Hospital, Surabaya, Indonesia; 8 Tyumen State Medical University, Tyumen, Russian Federation; 9 Industrial University, Tyumen, Russian Federation; 10 Faculty of Nursing, HRH Princess Chulabhorn College of Medical Science, Chulabhorn Royal Academy, Bangkok, Thailand; 11 Department of Neuroimmunology, National Institute of Neurology and Neurosurgery “Manuel Velasco Suarez” (INNN), 14269, Mexico City, Mexico; 12 Center for Transdisciplinary Research ,Department of Pharmacology, Saveetha Dental College, Saveetha Institute of Medical and Technical Science, Saveetha University, Chennai, India; 13 Cellular and Molecular Research Center, School of Medicine, Tehran University of Medical Sciences, PO Box: 1417613151, Tehran, Iran

**Keywords:** Mesenchymal stromal cell, Autoimmune diseases, Stem cell therapy, Clinical application

## Abstract

The autoimmune diseases are associated with the host immune system, chronic inflammation, and immune reaction against self-antigens, which leads to the injury and failure of several tissues. The onset of autoimmune diseases is related to unbalanced immune homeostasis. Mesenchymal stem cells (MSCs) are multipotent cells which have capability to self-renew and differentiate into various cell types that exert a critical role in immunomodulation and regenerative therapy. Under the certain condition in vitro, MSCs are able to differentiate into multiple lineage such as osteoblasts, adipocytes, and neuron-like cells. Consequently, MSCs have a valuable application in cell treatment. Accordingly, in this review we present the last observations of researches on different MSCs and their efficiency and feasibility in the clinical treatment of several autoimmune disorders including rheumatoid arthritis, type 1 diabetes, multiple sclerosis, systemic lupus erythematosus, inflammatory bowel disease, autoimmune liver disease, and Sjogren’s syndrome.

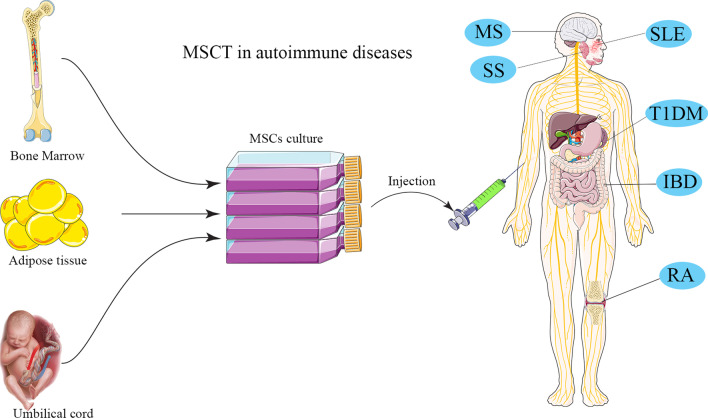

## Introduction

Autoimmune disorders include several chronic diseases which are frequently considered as organ-specific and systemic [1–3]. These diseases mainly occur because of the malfunction of immune system which mistakenly attack to own body’ cells and tissues [4–6]. Approximately 8–10% of the population is affected by autoimmune disorders which cause serious impairment, high mortality rate, and medical costs [7].

In recent years, stem cell-based therapy is progressively used as a therapeutic approach for various diseases such as autoimmune diseases. Stem cell transplantation, conventionally used for hematopoietic disorders, however, it is now established for the treatment of nonhematologic diseases [8–11]. The pivotal discovery of stem cells has provided a potential opportunity for accelerating tissue regeneration through switching damaged cells in paracrine and juxtacrine signaling modes. Mesenchymal stem/stromal cells (MSCs) display considerable trans-differentiation features into multiple lineages after implantation [12–14]. In order to utilize them in the clinical studies, it is obligatory to culture separated MSCs in vitro. Because of their ease collection procedure, existence in various tissues, differentiation into various cell lineages, and high proliferation rate, MSCs are more applied in stem cell therapy compared to the other stem cell types [15, 16]. The results of studies have demonstrated that MSCs can inhibit the proliferation and function of T lymphocytes, decrease the concentrations of tumor necrosis factor α (TNF-α), increase regulatory T (Treg) cells, regulate the expression of inflammatory mediators, and ameliorate bone injury [17–19]. The immunosuppressive and regenerative properties of MSCs show their great therapeutic ability in severe autoimmune disorders.

Considering these advantages, we provided a review of recent clinical studies which considered the efficiency of MSCs in autoimmune diseases including rheumatoid arthritis, type 1 diabetes, multiple sclerosis, systemic lupus erythematosus, inflammatory bowel disease, autoimmune liver disease, and Sjogren’s syndrome.

## Mesenchymal stem cell

According to the current evidence, MSCs are spindle-shaped and resemble fibroblasts that can be isolated from a variety of sources such as umbilical cord (UC), Wharton’s jelly (WJ), adipose tissue, bone marrow (BM), teeth and menstrual fluid [15, 20]. The MSCs originally explained by Friendenstein et al. in 1966 as bone forming cells in BM; nevertheless, they are usually named MSCs because they present adult stem cell multipotency [21]. They can differentiate to endothelial cells [22], cardiomyocytes [23], cartilage, bone and other connective tissues at the single cell level in vitro [24]. The International Society of Cellular Therapy (ISCT) suggests three criteria to describe MSCs. First, these cells are adherent to plastic once cultured in tissue flasks under standard conditions. Second, they express a variety of markers include CD73, CD90, and CD105, but lack CD45, CD34, CD14/CD11b, CD79α/CD19, and HLA-DR, and finally, the cells can differentiate into osteoblasts, adipocytes, and chondroblasts in vitro [25]. In addition, MSCs exert immunosuppressive abilities via their paracrine properties and communication with various immune cells and display low level of human leukocyte antigen (HLA) I, and rarely expression of HLA II. There is also a lack of co-stimulatory molecules such as CD40, CD40L, CD80, CD86 in MSCs which make them evading of T cell recognition [26–29]. It was shown that MSCs regulate their local environment, cellular communications, and the release of several factors, and participate in regeneration of tissue injury [30–34]. Indeed, they possess a homing capacity, can migrate into damaged tissues, and have the ability to differentiate into local components of damaged tissues and the capacity to release growth factors, cytokines, and chemokines, which improve tissue regeneration [35, 36].

### Clinical applications of mesenchymal stem cells

**Fig. 1:**
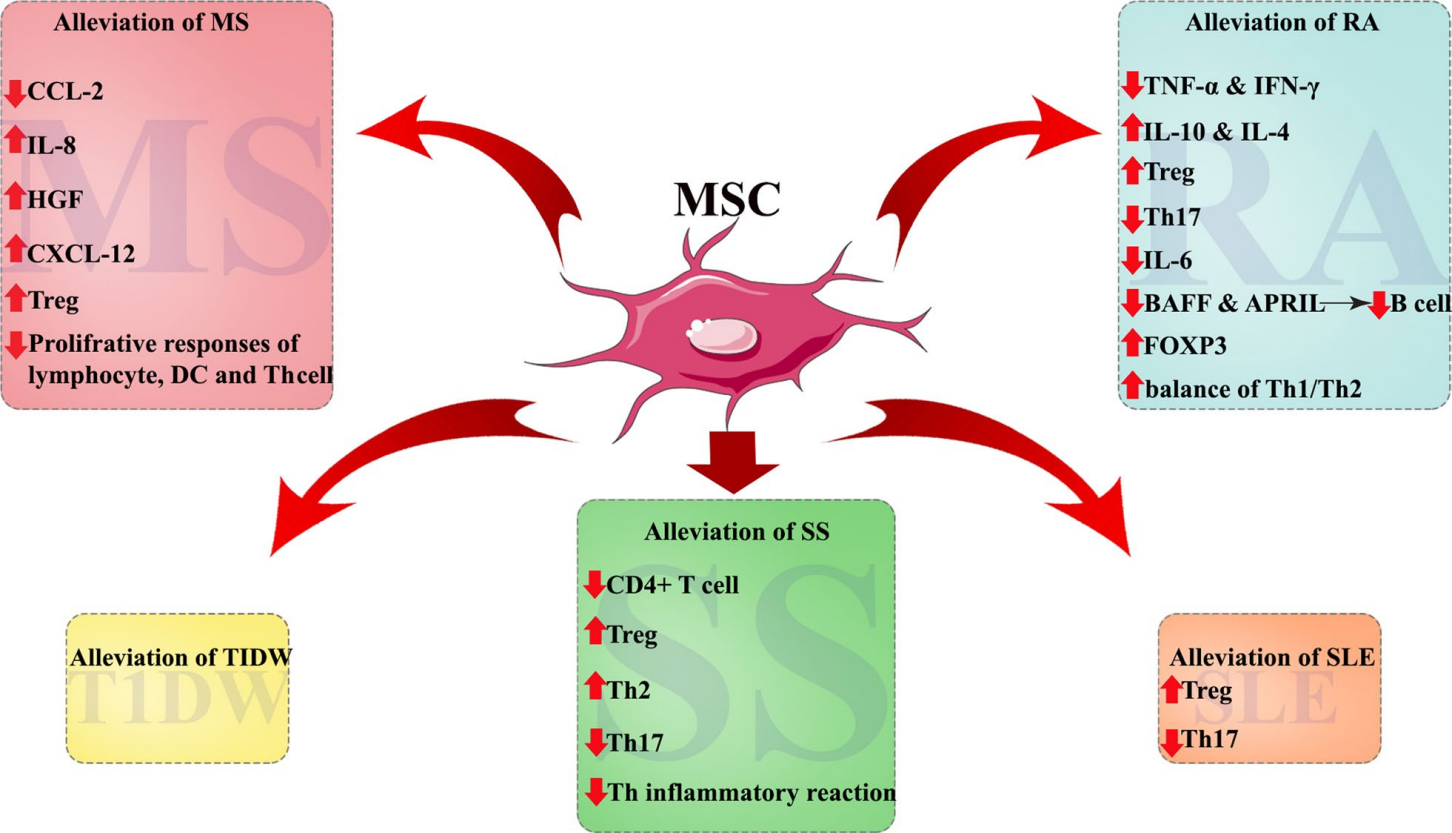
Clinical application of MSCs from different sources in treatment of autoimmune diseases. RA, rheumatoid arthritis; T1DM, type 1 diabetes mellitus; MS, multiple sclerosis; SLE, systemic lupus erythematosus; IBD, inflammatory bowel disease; SS, Sjogren’s syndrome

As mentioned in Table 1, many studies have evaluated the potential contribution of MSCs in treatment of various autoimmune diseases, which are discussed in the following sections (Fig. 1).

**Table 1:** Clinical application of mesenchymal stem cells in autoimmune diseases

Disease	Infusion method	MSC source	Enrollment number		Cell mass	Outcome	NCT number		Reference	
RA	I.V	Autologous BM-MSC	9		1 × 10^6^/kg	Clinical efficacy		NCT03333681		[45]
RA	I.V	Autologous BM-MSC	13		1 × 10^6^/kg	Reduction of B cells response		NCT03333681		[50]
RA	I.V	Autologous BM-MSC	13		1 × 10^6^/kg	Immunomodulatory effects of MSCT		NCT03333681		[53]
RA	Intra-articular knee	Autologous BM-MSC	30		40 × 10^6^/joint	Clinical efficacy		NCT01873625		[98]
RA	I.V	Allogeneic AD-MSC	20		1 × 10^6^/kg	Clinical efficacy		NCT01663116		[46]
			20		2 × 10^6^/kg					
			6		4 × 10^6^/kg					
RA	I.V	hUC-MSC with IFN-γ	63		1 × 10^6^/kg	Clinical efficacy		Unknown		[47]
RA	I.V	hUC-MSC	105		1 × 10^6^/kg	Clinical efficacy/IFN-γ levels predicts the therapeutic effects of MSCs		Unknown		[48]
RA	I.V	hUC-MSC	64		2 × 10^7^ cell	UC-MSC cells + DMARDs therapy can be a safe, effective and feasible		NCT01547091		[49]
RA	I.V	hUC-MSC	172		4 × 10^7^ cell	UC-MSC cells + DMARDs provide safe, and persistent clinical benefits		NCT01547091		[51]
RA	I.V	hUC-MSC with LG	119		4 × 10^7^ cell	LG + UC-MSCs can improve the curative effect of RA patients		NCT01547091		[99]
RA	I.V	hUC-MSC	3		2.5 × 10^7^cell	Clinical efficacy		NCT02221258		[52]
			3		5 × 10^7^ cell					
			3		1 × 10^8^ cell					
T1DM	I.V	hUC-MSC	53		1 × 10^6^/kg	Clinical efficacy		Unknown		[55]
T1DM	I.V	hUC-MSC	42		1.1 × 10^6^/kg	Clinical efficacy		NCT01374854		[58]
		BM-MNC			106.8 × 10^6^/kg					
T1DM	I.V	AD-ASC + Vit D	9		1 × 10^6^/kg	Clinical efficacy		NCT03920397		[56]
T1DM	I.V	AD-ASC + Vit D	13		67.37 ± 7.65 × 10^6^cells	Clinical efficacy		NCT03920397		[57]
T1DM	I.V	Autologous BM-MSC	20		2.1–3.6 × 10^6^/kg	Clinical efficacy		NCT01068951		[59]
T1DM	I.A	IS-AD-MSC + autologous BM-HSC	10		5.3 × 10^6^/ml	Autologous IS-AD-MSC + BM-HSC co-infusion offers better long-term control of hyperglycemia		Unknown		[60]
		IS-AD-MSC + allogenic BM-HSC			5.1 × 10^6^/ml					
T1DM	I.A	IS-AD-MSC plus BM-HSC	10		2.7 × 10^4^/kg	Clinical efficacy		Unknown		[100]
					60.55 × 10^6^/kg					
T1DM	I.A	IS-AD-MSC plus BM-HSC	11		Unknown	Clinical efficacy		Unknown		[101]
T1DM	I.V	WJ-MSC	29		2.6 ± 1.2 × 10^7^ cells	Clinical efficacy/restoration of function islet β cells		Unknown		[61]
MS	I.V	Autologous BM-MSC	10		1–2 × 10^6^/kg	Clinical efficacy		NCT00395200		[68]
MS	I.T	Autologous BM-MSC	10		110 ± 23.1 × 10^6^/cells	Clinical efficacy		NCT01895439		[72]
MS	I.V	Autologous BM-MSC	10		1.6 × 10^6^/kg	Clinical efficacy		NCT00395200		[69]
MS	I.T	Autologous BM-MSC	10		8.73 × 10^6^cells	Feasibility of autologous MSC for treatment of MS patients		Unknown		[102]
MS	I.T	Autologous BM-MSC	10		3–5 × 10^7^cells	Clinical but not radiological efficacy		Unknown		[103]
MS	I.T	Autologous BM-MSC	25		29.5 × 10^6^cells	Safe and feasible therapeutic approach		Unknown		[104]
MS	I.T & I.V	Autologous BM-MSC	15		2.5 × 10^6^cells	Clinically feasible and relatively safe procedure and induces immediate immunomodulatory effects		NCT00781872		[70]
MS	I.T & I.V	Autologous BM-MSC	48		1 × 10^6^/kg	Clinical efficacy/I.T administration was efficacious than the I.V		NCT02166021		[64]
MS	I.V	Autologous AD-MSC	10		1 × 10^6^/kg	Safe and feasible		NCT01056471		[71]
			9		4 × 10^6^/kg					
MS	I.V	hUC-MSC	20		20 × 10^6^/cells	Safe and feasible		NCT02034188		[65]
MS	I.V	hUC-MSC	23		4 × 10^6^/kg	High potential for hUC-MSC treatment of MS		Unknown		[105]
MS	I.V	Placenta-MSC	16		15 × 10^7^/cells	Safe and well tolerated in patients with MS		Unknown		[73]
					6 × 10^8^/cells					
MS	I.T	MSC-NP	20		5.3 × 10^6^ to 1 × 10^7^ cells	Clinical efficacy		NCT01933802		[66]
MS	I.T	MSC-NP	18		9.4 × 10^6^cells	Clinical efficacy		NCT01933802		[67]
SLE	I.V	BM-MSC	58		1 × 10^6^/kg	Clinical efficacy		NCT00698191		[106]
SLE	I.V	BM-MSC	15		1 × 10^6^/kg	Clinical efficacy		NCT00698191		[107]
SLE	I.V	BM-MSC	4		≥ 1 × 10^6^/kg	Clinical efficacy		NCT00698191		[108]
SLE	I.V	hUC-MSC	178		1 × 10^6^/kg	Safe and feasible		NCT00698191		[109]
SLE	I.V	hUC-MSC	40		Unknown	16 patients had no clinical response		NCT01741857		[80]
						Seven patients relapse after 6 months				
SLE	I.V	hUC-MSC	79		1 × 10^6^/kg	UC-MSCs suppressed T cell proliferation in lupus patients by secreting IDO		NCT01741857		[82]
SLE	I.V	hUC-MSC	18		2.8 × 10^8^/cells	hUC-MSC has no apparent additional effect over and above standard immunosuppression		NCT01539902		[76]
SLE	I.V	BM-MSC	81		1 × 10^6^/kg	Clinical efficacy		Unknown		[110]
		hUC-MSC								
SLE	I.V	BM-MSC	35		1 × 10^6^/kg	Clinical efficacy		NCT00698191		[77]
		hUC-MSC								
SLE	I.V	hUC-MSC	16		1 × 10^6^/kg	Clinical efficacy		NCT00698191		[81]
SLE	I.V	BM-MSC	87		1 × 10^6^/kg	Clinical efficacy		Unknown		[111]
		hUC-MSC								
IBD	I.L	AD-MSC	5		3–30 × 10^6^/cells	Safe and feasible		Unknown		[112]
IBD	I.L	AD-MSC	107		12 × 10^7^/cells	Clinical efficacy		NCT01541579		[86]
IBD	I.L	Autologous AD-MSC	5		Unknown	Safe and feasible		Unknown		[113]
IBD	I.L	AD-MSC	24		2 × 10^7^/cells	Safe and feasible		NCT01372969		[114]
IBD	I.V	Autologous BM-MSC	4		2 × 10^6^/kg	Clinical efficacy		NCT01659762		[115]
			4		5 × 10^6^/kg					
			4		10 × 10^6^/kg					
IBD	I.V	Autologous BM-MSC	10		1–2 × 10^6^/kg	Safe and feasible		NTR1360		[116]
IBD	I.V	BM-MSC	16		2 × 10^6^/kg	Clinical efficacy		NCT01090817		[3]
IBD	I.V	hUC-MSC	41		1 × 10^6^/kg	Clinical efficacy		NCT02445547		[88]
IBD	I.L	BM-MSC	5		1 × 10^7^/cells	Clinical efficacy		NCT01144962		[89]
			5		3 × 10^7^/cells					
			5		9 × 10^7^/cells					
IBD	I.V	BM-MSC	30		3 × 10^6^/kg	Safe and effective		Unknown		[117]
IBD	I.L	BM-MSC	5		1 × 10^7^/cells	Clinical efficacy		NCT01144962		[90]
			5		3 × 10^7^/cells					
			5		9 × 10^7^/cells					
SS	I.V	hUC-MSC	24		1 × 10^6^/kg	Safe and effective		NCT00953485		[93]

RA, rheumatoid arthritis; T1DM, type 1 diabetes mellitus; MS, multiple sclerosis; SLE, systemic lupus erythematosus; IBD, inflammatory bowel disease; SS, Sjogren’s syndrome; I.V, intravenous; I.T, intrathecal; I.L, intralesional; I.A, interatrial; MSC, mesenchymal stem cell; BM, bone marrow; hUC, human umbilical cord; AD, adipose tissue; ASC, adipose stem cell; IS, insulin-secreting

### Rheumatoid arthritis

**Fig. 2:**
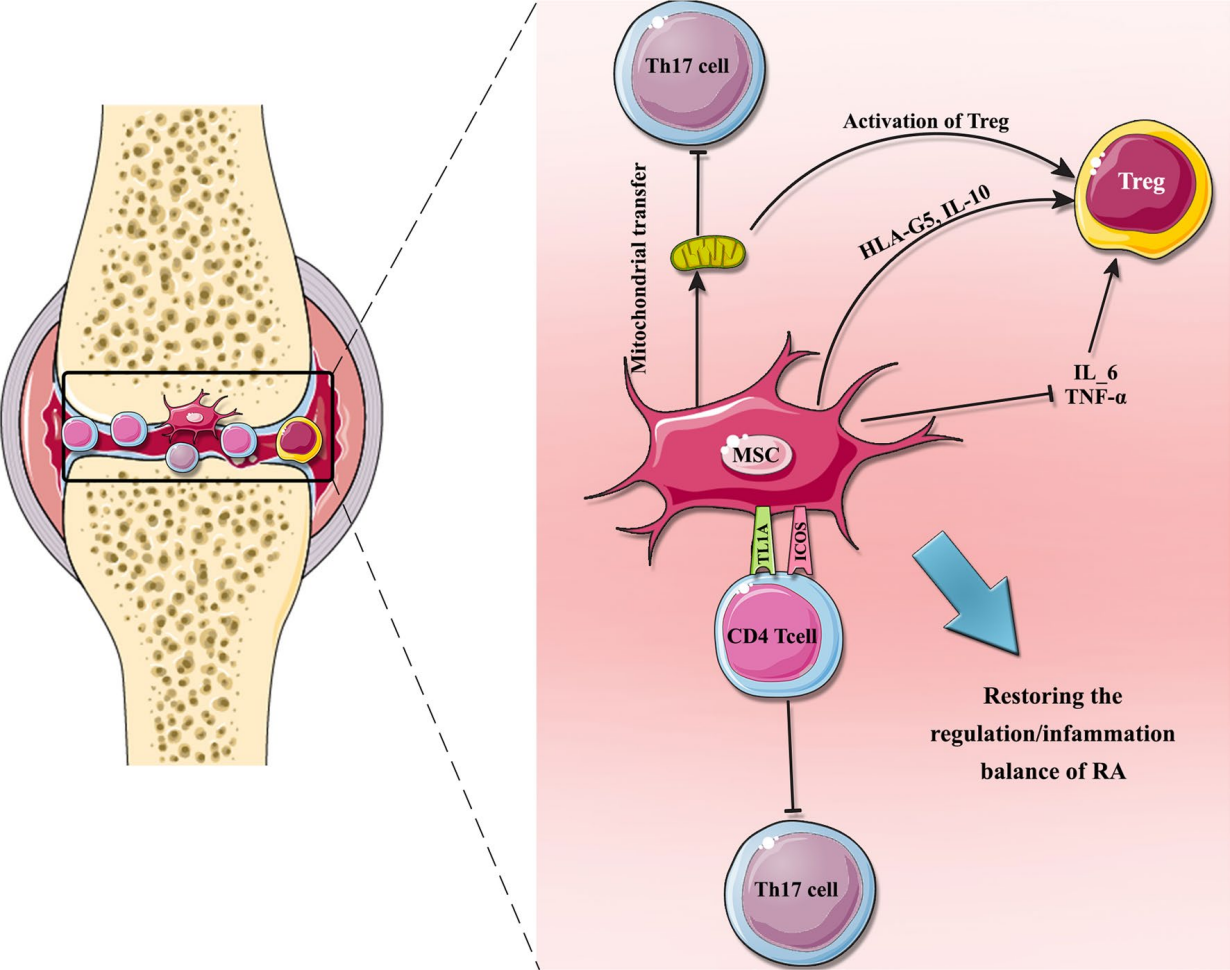
MSCs ameliorate RA by regulating T cells. MSCs can regulate the balance of T cells by homing to the articular cavity and releasing various cytokines that elevate the anti-inflammatory activity of the environment. T cells are also regulated by the transfer of mitochondria from MSCs. In addition, Th17 cells differentiation suppressed by costimulatory molecules ICOS (inducible costimulatory), and TL1A (TNF-like ligand 1A)

Rheumatoid arthritis (RA) is the most common systemic autoimmune disease worldwide. It is characterized by articular inflammation, synovial membrane hyperplasia as well as progressive joint damage, cartilage and bone destruction which worsening disability over time. The onset of RA is related to unbalanced immune homeostasis, most considerably, between T helper 17 (Th17) and Tregs lead to the activation of autoreactive immune cells which attack collagen-rich joint regions [37, 38]. Patients with RA have also elevated risk for developing cardiovascular disease in comparison with general population [39]. To treat RA, conventional drugs including nonsteroidal anti-inflammatory drugs (NSAIDs), corticosteroids, slow-acting anti-rheumatic drugs (SAARDs), and disease-modifying antirheumatic drugs (DMARDs) are recommended in accordance with the severity of pathology [37, 40]. Methotrexate (MTX) remains the primary preferred antirheumatic drug and is the best candidate for RA therapy which ordinarily recommended to these patients [41]. As the mentioned drugs often cause liver injury, gastrointestinal injury, kidney side effects, BM suppression, and psychological disorders, the search for new innovative therapeutic approaches is an important issue [41, 42]. Studies have shown that MSCs decrease the production of the proinflammatory cytokines such as TNF-α and interferon-γ (IFN-γ), whereas simultaneously increases secretion of anti-inflammatory cytokines like interleukin-10 (IL-10) and IL-4 which play a major role in tissue regeneration [43]. These features suggest that MSCs could be an emerging therapeutic option in treatment of RA. The results of studies have also indicated that MSCs can ameliorate the RA through different mechanisms such as suppression of Th17 cells, reduction of inflammatory cytokines, and up-regulation of Treg cells (Fig. 2) [44].

In a clinical report by Ghoryani et al. [45], autologous BM-MSCs were applied for treatment of patients with refractory RA. All nine participants intravenously received 1 × 10^6^ autologous BM-MSCs/kg. After MSCT, a major decrease in Th17 percentage and a significant increase in regulatory T cells were observed. Furthermore, disease activity score 28-erythrocyte sedimentation rate (DAS28-ESR) and visual analogue scale (VAS) were significantly reduced, but no noticeable difference was detected for serum C-reactive protein (CRP) and anti-cyclic citrullinated peptide (anti-CCP) levels after the intervention. These findings propose that autologous BM-MSCs can ameliorate refractory RA. In 2017, a multicenter, single blind, randomized phase Ib/IIa clinical trial using adipose-derived MSCs (AD-MSCs) in 53 patients with RA was reported [46]. Three groups were enrolled in this study which intravenously injected with different doses of AD-MSCs (1, 2, and 4 × 10^6^ cells per kg of body weight). Overall, 141 adverse effects were observed in these participants that 133 were of moderate intensity (94%), and there were no life threatening effects, (grade 4) or deaths. The clinical advantage achieved in RA patients diminished or fluctuated after 12 weeks of cell administration, demonstrating that it is vital to have a repeat transplant. Moreover, Xu et al. [47] demonstrated that IFN-γ is an important mediator in determining the impact of MSCs in RA therapy. They showed that MSC + IFN-γ combination therapy synergistically augments the potential of MSC therapy in participants with RA without any adverse events during 1 year follow up.

Yang and coworkers additionally supported the therapeutic effects of UC-MSCs in patients with persistently active RA [48]. Their results showed that the percentage of Tregs and Th17 was increased and decreased, respectively. Also the concentrations of IL-6 and TNF-α were reduced and the levels of IL-10 were increased. These findings suggest that MSCs could play main roles in regulating immune homeostasis. Furthermore, serum IFN-γ levels predict the therapeutic effect of MSCT; a transient increase in serum IFN-γ (> 2 pg/ml) levels was observed before changes in levels of IL-6, TNF-α, IL-10, and the Treg/Th17 ratio. Wang and colleagues [49] performed another clinical phase I/II trial included 64 refractory RA patients. These patients received 40 mL of UC-MSC product (2 × 10^7^ cells/20 mL) intravenously after 100 mL normal saline infusion. The results showed that Health index (HAQ) and DAS28 reduced after intervention. Also, serological markers and symptoms had improved, and there were no serious adverse events.

A study conducted by Gowhari et al. in 2020 has also examined the effect of autologous MSCs in thirteen patients with refractory RA. The results showed that MSCT suppressed B lymphocytes via decreasing the concentration of B-cell activating factor (BAFF) and a proliferation-inducing ligand (APRIL) cytokines as well as reducing the expression of their receptors on the B-cell surface. Their findings demonstrated a substantial decrease in the plasma levels of BAFF and APRIL following MSC administration, suggesting the significant effects of MSCs on humoral responses. These outcomes proposed that BAFF could be a hopeful candidate for subsequent evaluation of the pathogenesis of RA [50]. According to Wang et al. [51] clinical study, intravenous administration of UC-MSCs (4 × 10^7^ cells) in 172 individuals with RA ameliorates the disease which was generally associated with reduced expression levels of several inflammatory cytokines and chemokines. In addition, the percentage of Treg and the IL-4 producing Th2 was increased. No serious adverse events were also observed during and after infusion of UC-MSCs. The main goal from the study was that DMARDs plus UC-MSCs infusion was harmless and effective in reducing disease activity in patients with refractory RA than controls receiving DMARDs plus medium without UC-MSCs. Similarly, in a clinical phase Ia study by PARK et al., UC-MSCs were applied for treatment of RA. The patients were intravenously injected with 2.5 × 10^7^, 5 × 10^7^, or 1 × 10^8^ cells of UC-MSCs. Their findings illustrated enhanced symptoms and serological marker in all of the patients. No major adverse events were observed up to 4 weeks after each infusion of UC-MSCs [52].

Recently, Ghoryani et al. [53] indicated that intravenous injection of 1 × 10^6^ autologous BM-MSCs per kg into 13 patients with refractory RA significantly up-regulates the gene expression of forkhead box P3 (FOXP3) in peripheral blood mononuclear cells (PBMCs) after 1 year. Their data also presented the appropriate immunomodulatory potential of the BM-MSCs on Tregs in RA patients, and authors hypothesized that the elevation in the number of MSCs could support their immunosuppressive properties in these patients.

Summarily, these data exhibited that MSCT could be a hopeful, safe, and impressive option for the clinical therapy of RA, considerably ameliorate the clinical symptoms of patients, and prevent disease progression.

### Type 1 diabetes

Type 1 diabetes mellitus (T1DM) is a group of autoimmune diseases wherein autoreactive immune cells, especially CD4^+^ T cells, target pancreatic beta cells and cause complete insulin deficiency [54]. Increasing evidence has shown the therapeutic advantages of MSCs in clinical treatment of T1DM. For instance, Lu et al. [55] performed a nonrandomized, open-label, parallel controlled clinical report in which 1 × 10^6^/kg allogeneic UC-MSCs were infused to 53 patients with T1DM, followed by an another dose after 3 months. They have found that the complete remission rate was 40.7% during 1-year follow-up. They have also showed that the level of postprandial C-peptide was obviously elevated between the adult-onset T1DM, however, its alteration was not obviously different among the juvenile-onset T1DM. No transplant-related severe adverse effects were observed.

In a recent pilot study, patients with T1DM were administered by one dose of 1 × 10^6^/kg allogenic adipose tissue-derived stromal/stem cells (ASCs) and cholecalciferol 2000 UI/day for 6 months and compared with controls [56]. The authors declared that the glycosylated hemoglobin (HbA1C) values were noticeably improved without a remarkable elevation in insulin dose/kg that might be followed by the up-regulation in basal insulin release reported in those patients. There were several side effects in these patients including transient headache, abdominal cramps, scotomas, thrombophlebitis, and mild local reactions. Nonetheless, the number of participants in this trial was too low and the follow-up period was short. Taken together, treatment with ASC was safe and caused few or transient adverse events. Recently, in a similar study conducted by Araujo et al. [57], 13 patients were transplanted with 1 × 10^6^ per kg allogenic ASCs and cholecalciferol 2000 UI/day for 3 months and compared with control group. This study also showed the efficacy and safety of allogenic ASCs in T1DM therapy. No serious side effects associated with ASCs were observed in these patients.

In another study conducted by Cai et al. [58], 42 patients with T1DM were randomized to receive UC-MSCs (1.1 × 10^6^/kg) plus 106.8 × 10^6^/kg autologous BM-mononuclear cell (MNCs). Within 1 year, C-peptide was elevated, HbA1c reduced, fasting glycemia decreased, and daily insulin requirements decreased. Based on these results, UC-MSC and BM-MNC were safe and led to the improvement of metabolic measures in patients with T1DM. Carlsson et al. demonstrated that the autologous MSCT in new onset T1DM patients could be an efficient and safe approach to interfere with the process of T1DM and protect or restore pancreatic β cells function [59].

In the other study, 20 individuals divided into two groups; group 1 received autologous insulin-secreting AD-MSC (IS-AD-MSC) + BM-derived hematopoietic stem cell (BM-HSC) and group 2 treated with allogenic IS-AD-MSC plus BM-HSC [60]. No serious effects were reported with continual progress in HbA1c and serum C-peptide in both groups with a reduction in glutamic acid decarboxylase antibodies and decrease in mean insulin requirement. Their observations illustrated that autologous IS-AD-MSC injection showed better response in patients than allogenic IS-AD-MSC infusion.

In 2013, a double-blind study was reported that used intravenous infusion of WJ-MSCs in 29 patients with T1DM [61]. There were no reported adverse events and both the HbA1c and C peptide were significantly improved during the follow-up period. These findings suggested that the infusion of WJ-MSCs is safe and feasible for the treatment of T1DM.

### Multiple sclerosis

Multiple sclerosis (MS) is a chronic autoimmune inflammatory disorder of the central nervous system (CNS). However, the exact pathophysiology of MS remains unclear. It is mainly concurred that autoreactive T cells, stimulated by either self-reactive or cross-reactive antigens, result in demyelination and progressive neurodegeneration of the CNS. In spite of the fact that available therapies like drugs help to the reduction of MS development or decrease disability in these patients, they lead to serious side effects and do not reverse the manifestations of MS [62, 63].

In 2020, a double-blind, randomized controlled trial was reported that used intrathecal (IT) and intravenous (IV) infusion of autologous BM-MSCs (1 × 10^6^/kg) for treatment of 48 patients with progressive MS [64]. The participants divided into three groups according to injection method (IT or IV) and received a single infusion of BM-MSCs or sham injections. Their findings demonstrated positive results (Expanded Disability Status Scale (EDSS) and magnetic resonance imaging (MRI)) in all predefined primary end points. No severe adverse effects were observed. However, they revealed that IT administration was more effective than the IV in several parameters of the disease. Despite the above mentioned, a larger phase III study is warranted to confirm these observations.

Furthermore, in a research by Riordan et al., 20 patients with MS was intravenously administrated with UC-MSCs [65]. The authors indicated that MS symptoms were considerably ameliorated by MSCT. Furthermore, EDSS scores as well as bladder, bowel, sexual dysfunction, and quality of life were improved. Also, MRI scans of the brain and the cervical spinal cord displayed inactive lesions and did not report any serious adverse events during or after intervention. However, headache or fatigue was noted as probably associated with the intervention.

In a phase I open-label clinical trial by Harris et al. [66], 20 patients with progressive MS were intrathecally injected with autologous BM-MSC-neural progenitors (NPs) every 12 weeks for a total of 3 doses (1 × 10^7^ cells per dose). Their observations demonstrated that intrathecal MSC-NPs intervention was safe and well tolerated. In addition, the results represented an improvement in EDSS, muscle strength, and bladder function, respectively, following intrathecal MSC-NP administration. No severe adverse effects or hospitalizations related to intrathecal MSC-NP treatment were observed. The authors also hypothesized that a larger phase II placebo-controlled study is warranted to identify efficacy of intrathecal MSC-NP intervention in patients with MS.

In another long-term phase I clinical study which was conducted by Harris et al. [67], 20 patients with progressive MS were enrolled. The patients from 2014 to 2016 received three times IT injections of autologous MSC-NPs at an average dose of 9.4 × 10^6^ cells (target dose was 1.0 × 10^7^ cells). The results exhibited improvement in EDSS, and the timed 25-foot walk (T25FW). Furthermore, CSF investigation showed a decline in C–C motif chemokine ligand 2 (CCL2) and an elevation in IL-8, hepatocyte growth factor (HGF), and C-X-C motif chemokine ligand 12 (CXCL12) after intervention. There were no serious adverse events related to IT-MSC-NP treatment. Nevertheless, the number of participants of this study was small, and there was no blinding and placebo group for comparison. Similarly, Connick and colleagues represented the improvement in MS patients after MSC treatment [68]. In another study by Connick et al. [69], MSCT ameliorated patients with progressive MS. A single dose of 1.6 × 10^6^ per kg autologous BM-MSCs were intravenously administrated into the patients. They did not find any severe adverse effects. The results showed an enhancement in EDSS, log of minimum angle of resolution (logMAR) visual acuity, and low contrast visual acuity. They did not recognize any considerable effects on color vision, visual fields, macular volume, retinal nerve fiber layer thickness, or optic nerve magnetization transfer ratio. Taken together, the results of this intervention showed their neuroprotective effects in MS patients. In a study by Karussis et al., 15 MS patients were intrathecally received 2.5 × 10^6^ cells autologous BM-MSCs, also five of the total patients received intravenous infusion of BM-MSCs (2.5 × 10^6^ cells) [70]. The EDSS analysis represented positive results in MSC treatment. No serious adverse effects were showed during follow-up. Immunological analysis exhibited an increase in Tregs, reduction of proliferative responses of lymphocytes, and dendritic cells and a similar reduction in the number of Th cells. Interestingly, the quantitative analysis on MRI showed dissemination of MSCs from the infusion site to the ventricles of the CNS.

Fernandez et al. [71] reported a triple-blind, placebo-controlled study that involved 30 patients with MS who divided into two groups. Group 1 injected with low-dose (1 × 10^6^ cells/kg) and group 2 received high-dose (4 × 10^6^ cells/kg) autologous AD-MSCs and followed for 1 year. Evidence for this treatment showed an inconclusive trend of efficacy. There was just one major untoward effect in the MSC therapy.

Moreover, in a phase I/IIa clinical trial, ten patients with MS were injected of autologous BM-MSCs conditioned medium (MSC-CM) via the intrathecal route for the first time [72]. The results showed a general trend of enhancement in all the analysis, but the lesion volume elevated considerably. No serious adverse effects were reported during study. In addition, they demonstrated an association between a reduced white matter lesion number at baseline and higher IL-6, IL-8, and VEGF in MSC-CM content.

Another randomized, placebo clinical study which was conducted by Lublin and colleagues [73] applied placenta mesenchymal-like cells for treatment of 16 patients suffering from relapsing–remitting or progressive MS. Two groups participated in this trial who received low-dose (15 × 10^7^cells) and high-dose (6 × 10^8^cells) injection of these cells. According to the results, the safety and feasibility of this therapy were demonstrated in these patients. However, anaphylactoid reaction was seen as grade 1 side effects in one of the patients.

### Systemic lupus erythematosus

Systemic lupus erythematosus (SLE), characterized by the high production of nuclear autoantibodies, is a chronic inflammatory autoimmune disease, which result in antibody-antigen immune complexes deposition in various organs [74]. In addition to an imbalance of Th1/Th17/Tregs, it seems that the regulatory B cells (Bregs) have a key role in pathogenesis of the SLE [75].

Deng et al. [76] performed a prospective, randomized, double-blind clinical study with a total infusion dose of 2 × 10^8^ UC-MSCs in 18 patients with SLE. The results illustrated an improvement in renal function and decreased proteinuria, whereas serum albumin has elevated. In addition, other indices of SLE were improved. These comprised SLE Disease Activity Index (SLEDAI), British Isles Lupus Assessment Group (BILAG), anti-double-stranded DNA antibody (dsDNA) antibody and antinuclear antibody (ANA) titers and serum complement C3 and C4 concentrations. Four major adverse effects were also reported during study. Unfortunately, the study was abandoned when it had become apparent that the study would be unlikely to establish a positive treatment effect for UC-MSC.

Li et al. [77] have been shown that BM-MSCs could improve hematological abnormality and clinical remission in SLE patients with refractory cytopenia, which might be associated with increased Treg and decreased Th17.

It has also been declared that the soluble human leukocyte antigen-G (sHLA-G), a non-classical HLA class I molecule, is considerably up-regulated in serum of SLE patients along with the increase of Tregs following the administration of UC-MSCs which alleviate SLE [78]. Additionally, another important immunomodulatory effects of MSCs are related to IL-10 which induce secretion of HLA-G5 molecule [79].

Wang et al. [80] also performed a clinical trial that included 40 patients with active and refractory SLE. Their observations exhibited a significant decline in SLEDAI and BILAG scores as well as proteinuria, serum creatinine, and urea nitrogen. Additionally, serum concentration of albumin and complement amplified after UC-MSC infusion. No administration-related adverse events were showed and all participants tolerated the intervention well. However, seven patients relapsed 6 months after intervention, showing the requirement for a second treatment to avoid relapse. Likewise, Sun et al. [81] illustrated that the injection dose of UC-MSCs was directly associated with their efficacy. They also found that MSCT ameliorated disease activity, serologic changes, and stabilization of proinflammatory cytokines. In another study, the authors showed that allogeneic UC-MSCs mediate immunosuppression via suppression of T cell proliferation in SLE patients by releasing high levels of indoleamine 2, 3-dioxygenase (IDO) [82].

In addition, a study was conducted by Wang et al. [83] in 2016 to evaluate the safety of allogeneic UC-MSC therapy for refractory SLE patients. Nine patients were administrated intravenously at days 0 and 7 and followed up during 6 years. There were no adverse effects like fluster, headache, nausea, or vomit in these patients.

### Inflammatory bowel disease

Inflammatory bowel disease (IBD) is a chronic inflammatory gastrointestinal and autoimmune disease that includes ulcerative colitis (UC) and Crohn’s disease (CD). IBD is mostly resulted from inappropriate and ongoing immune response of genetically susceptible hosts to pathogenic organism [84].

Several studies have shown that therapeutic potential of MSCs in treatments of IBD could restore epithelial barrier integrity [85]. In a phase 3 clinical study by Panés et al. [86], 212 patients were injected intralesionally with 120 × 10^6^ allogeneic AD-MSCs. The results of the study revealed that the treatment group achieved combined remission in the intention-to-treat (ITT) and modified ITT populations at 24 weeks after treatment, showing the efficiency and safety of MSC therapy in CD. Eighteen of the total patients experienced treatment-related adverse events such as anal abscess and proctalgia. Philandrianos et al. [87] also reported that after administration of autologous adipose-derived stromal vascular fraction (ADSVF), perianal Crohn’s fistulas had clinically healed with complete re-epithelialization.

In a randomized controlled clinical trial conducted by Zhang et al., 82 patients were intravenously received UC-MSCs [88]. According to their findings, CD symptoms were remarkably ameliorated by MSC injection. CD activity index (CDAI), Harvey–Bradshaw index (HBI), and corticosteroid level were also improved. There were no further MSCT associated adverse events. In a phase 2 study, Forbes et al. exhibited that infusion of allogeneic MSCs improved CDAI and CD endoscopic index of severity (CDEIS) scores in patients with luminal CD refractory to biologic therapy [3].

In a long-term retrospective trial by Barnhoorn et al. [89], 21 participants with refractory CD were treated with 1 × 10^7^/kg BM-MSCs. The 4-year follow-up results exhibited that Crohn’s fistulas closure rates had clinically alleviated. In none of the participants anti-HLA antibodies could be identified 24 weeks and 4 years following MSCT. This long-term study displayed that MSCT is able to ameliorate fistulas in CD patients and recovered patients’ quality of life. Furthermore, any adverse events thought to be associated with MSCT. Molendijk et al. [90] described another clinical double-blind research included 21 patients who were distributed into three groups and given a single injections of 1 × 10^7^, 3 × 10^7^, and 9 × 10^7^ BM-MSCs. The results indicated that local treatment with 3 × 10^7^ MSCs could more efficiently promote healing of perianal fistulas.

Therefore, MSCT can be effective, feasible, and safe treatment method which noticeably increase fistulas closure rates, improve CDAI and CDEIS scores, and promote patients’ quality of life.

## Sjögren’s syndrome

Sjogren’s syndrome (SS) is one of the three most common autoimmune disorders in which lymphocytes infiltrate into salivary and lacrimal glands [91]. It is a multifaceted disorder and the hallmark characteristics include dry mouth and eyes, and joint pain [91, 92]. Due to their beneficial abilities in suppression the differentiation and proliferation of many immune cells, production of inflammatory factors, and secretion of antibodies, their injection has been used as a novel approach to treat SS.

In a study performed by Xu etal., 24 participants with SS were intravenously administrated with UC-MSCs [93]. They showed that SS manifestations were notably decreased, the Sjogren’s syndrome Disease Activity Index (SSDAI) and VAS were ameliorated, and salivary flow rate increased by MSCT. However, no serious adverse events occurred during or after MSC administration. The results revealed that the beneficial properties of MSCT in treatment of diseases were attributed to their immunomodulatory feature such as regulation of CD4^+^ T lymphocytes, up-regulation of Tregs and Th2 cells, and down-regulation of T17 and Tfh inflammatory reactions. In addition, they also exhibited a vital role of the stromal cell-derived factor-1/C-X-C chemokine receptor type 4 (SDF-1/CXCR4) axis in guiding MSC toward inflammation sites, to play inhibitory activities and improved the function of salivary glands.

### Autoimmune liver disease

Autoimmune liver disease (AILD) is one of the chronic renal conditions resulting from malfunction of the immune system that including autoimmune hepatitis (AIH), primary biliary cholangitis (PBC), and primary sclerosing cholangitis (PSC). The clinical symptoms of these conditions include: fatigue, reduced appetite, liver pain, and scleral icterus, and cause abnormal levels of liver function markers such as alanine aminotransferase (ALT), aspartate aminotransferase (AST), alkaline phosphatase (ALP), gamma-glutamyl transferase (GGT), IgM, IgG, and presence of autoantibodies in blood tests [94, 95].

In a pilot study conducted by Wang et al., seven patients with PBC were intravenously administrated with UC-MSCs (0.5 × 10^6^cells/kg) once every month on three times [96]. After MSCT, serum ALP and GGT values were meaningfully reduced, but no adverse events were observed during and after trials. Some of the common manifestations of PBC patients such as fatigue and pruritus were significantly ameliorated. These findings indicated that MSCT can reduce the severity of PBC and is safe and feasible procedure. In another study, ten patients with PBC were received a single dose of 3–5 × 10^5^cells/kg allogeneic BM-MSCs [97]. The results of this study demonstrated that the life quality of the participants was enhanced after MSCT. Liver biomarkers exhibited that the level of ALT, AST, GGT, IgM, and direct bilirubin remarkably reduced from baseline after intervention during the 12-month follow-up period. Furthermore, the level of Treg cells in the peripheral blood mononuclear cells of participants remarkably up-regulated, while the level of CD8 + T cells was decreased following the infusion of BM-MSCs which enhanced PBC. Their observation indicated that the levels of IL-10 were also increased, while no therapy-related side effects were reported.

To date, there were no clinical study to assess the effect of MSCs on another AILDs such as AIH and PSC.

## Conclusion and outlook

In recent years, MSCs have indicted notable implications in clinical trials and treatments of various autoimmune diseases because of their beneficial properties such as safe and easy obtaining procedure, high proliferation ability and multipotent differentiation capacity as well as anti-inflammatory and immunomodulatory properties, and regenerative potential. In addition to this, their low tumorigenic effects along with poor immunogenicity make these cells as an emerging option in clinical treatment of various disorders and regeneration therapy. According to the clinical trials explained in our review, the repeated administration of MSCs have more effects in comparison with a single infusion. The MSCs were applied intravenously in most of the studies and the injection dosage was mainly between 1 × 10^6^ and 1 × 10^8^ cells/kg.

Furthermore, no remarkable association was found between the MSCT and occurrence of tumor and infection. However, there is still a lack understanding of the mechanisms through which the MSCT ameliorate the various autoimmune diseases which can facilitate the MSC modification and enhance their future clinical use.

## Data Availability

Not applicable.

## Abbreviations

AILD: Autoimmune liver disease
AIH: Autoimmune hepatitis
ALT: Alanine aminotransferase
AST: Aspartate aminotransferase
ALP: Alkaline phosphatase
GGT: Gamma-glutamyl transferase
ANA: Antinuclear antibody
ADSVF: Adipose-derived stromal vascular fraction
ASCs: Adipose tissue-derived stromal/stem cells
AD-MSCs: Adipose-derived-mesenchymal stem cells
Anti-CCP: Anti-cyclic citrullinated peptide
APRIL: A proliferation-inducing ligand
BAFF: B-cell activating factor
BM: Bone marrow
BILAG: British Isles Lupus Assessment Group
BM-HSC: Bone marrow-derived hematopoietic stem cell
Bregs: Regulatory B cells
CD: Crohn’s disease
CNS: Central nervous system
CXCL12: C-X-C motif chemokine ligand 12
CRP: C-reactive protein
CCL2: C-C motif chemokine ligand 2
CDAI: Crohn’s disease activity index
CDEIS: Crohn’s disease endoscopic index of severity
CXCR4: C-X-C chemokine receptor type 4
DsDNA: Anti-double-stranded DNA antibody
DMARDs: Disease-modifying antirheumatic drugs
EDSS: Expanded Disability Status Scale
FOXP3: Forkhead box P3
HbA1C: Glycosylated hemoglobin
HBI: Harvey–Bradshaw index
HGF: Hepatocyte growth factor
HLA: Human leukocyte antigen
IBD: Inflammatory bowel disease
IDO: Indoleamine 2, 3-dioxygenase
IL-10: Interleukin-10
IFN-γ: Interferon-γ
IV: Intravenous
IT: Intrathecal
IS-AD-MSC: Autologous insulin-secreting adipose-derived mesenchymal stem cells
ISCT: International Society of Cellular Therapy
LogMAR: Log of minimum angle of resolution
MSCs: Mesenchymal stem cells
MNCs: Mononuclear cell MNCs
MS: Multiple sclerosis
MRI: Magnetic resonance imaging
MTX: Methotrexate
NPs: Neural progenitors
NSAIDs: Nonsteroidal anti-inflammatory drugs
PBC: Primary biliary cholangitis
PSC: Primary sclerosing cholangitis
PBMCs: Peripheral blood mononuclear cells
RA: Rheumatoid arthritis
SAARDs: Slow-acting anti-rheumatic drugs
sHLA-G: Soluble human leukocyte antigen-G
SLE: Systemic lupus erythematosus
SLEDAI: Systemic lupus erythematosus disease activity index
SS: Sjogren’s syndrome
SSDAI: Sjogren’s syndrome disease activity index
SDF-1: Stromal cell-derived factor-1
TNF-α: Tumor necrosis factor α
Th17: T helper 17
T1DM: Type 1 diabetes mellitus
T25FW: Timed 25-foot walk
Treg: Regulatory T cells
UC: Umbilical cord
UC: Ulcerative colitis
WJ: Wharton’s jelly
